# Towards Precision Geromedicine in Singapore

**DOI:** 10.1007/s11357-025-01686-7

**Published:** 2025-05-08

**Authors:** Jonas John Posko Amalaraj, Louis Island, Jane Yu Ying Ong, Laureen Wang, Jose Hans M. Valderas, Michael Dunn, Yap Seng Chong, Johannes Meij, Andrea B. Maier

**Affiliations:** 1 NUS Academy for Healthy Longevity, Yong Loo Lin School of Medicine, National University of Singapore (NUS), Singapore, 117456 Singapore; 2 Well Programme, Alexandra Hospital, National University Health System (NUHS), 378 Alexandra Rd, Singapore, 159964 Singapore; 3 Centre for Research in Health Systems Performance (CRiHSP), National University of Singapore (NUS), Singapore, 117456 Singapore; 4 Centre for Biomedical Ethics, Yong Loo Lin School of Medicine, National University of Singapore, Singapore, 117597 Singapore; 5 Yong Loo Lin School of Medicine, National University of Singapore, Singapore, 117597 Singapore; 6 Institute for Human Development and Potential, Agency for Science, Technology and Research (A*STAR), Singapore, Singapore; 7 Department of Obstetrics & Gynaecology, National University Health System, Singapore, Singapore; 8 Outpatients Division, Amsterdam University Medical Center, University of Amsterdam, Meibergdreef 9, Amsterdam, The Netherlands; 9 Department of Human Movement Sciences, @AgeAmsterdam, Faculty of Behavioural and Movement Sciences, Vrije Universiteit Amsterdam, Amsterdam Movement Sciences, Van Der Boechorststraat 7, 1081 BT Amsterdam, The Netherlands; 10 Healthy Longevity Translational Research Programme, Yong Loo Lin School of Medicine, National University of Singapore, Singapore, 117597, Singapore

**Keywords:** Ageing, Healthy ageing, Longevity, Primary health care, Healthy life expectancy

## Abstract

Since the discovery that ageing is a modifiable process in animal models, significant advancements in geroscience have led to the emergence of the field of Precision Geromedicine, which aims to optimise health and healthspan by targeting ageing-related processes. Ageing-related diseases (ARDs), accounting for 80% of Singapore’s disease burden in 2019, are on the rise as the nation approaches the “super-aged” status by 2030. In response, Singapore is reshaping its healthcare system to focus on healthy ageing, as seen in the launch of the Healthier SG initiative in 2023, which empowers citizens to manage their health proactively with support from over 1800 private general practices. Additionally, Singapore is investing in geroscience to build the foundations of Precision Geromedicine, aiming to integrate gerodiagnostics and gerotherapeutics into clinical practice. Leveraging its robust healthcare system, digital infrastructure, and socio-political stability, Singapore is well-positioned to become a model for addressing ARDs amidst global demographic shifts.

## Introduction

The recognition that ageing is a modifiable process dates back to 1935 when caloric restriction was shown to extend lifespan in animal models [1]. This discovery spurred efforts in the 1960 s to identify biomarkers of ageing, accelerating the field of geroscience by the late twentieth century [2]. The geroscience hypothesis posits that ageing-related diseases (ARDs) can be prevented by targeting key biological pathways associated with ageing, often described through the hallmarks of ageing [3]. Parallel to these developments, clinical translation to healthy longevity medicine has evolved, culminating in the emergence of Precision Geromedicine, a specialised medical field built on geroscience principles. Precision Geromedicine aims to optimise health and extend healthspan by targeting ageing processes throughout the lifespan [4, 5]. While both Healthy Longevity Medicine and Precision Geromedicine refer to the same core concept, the former is designed for a broader, lay audience, and the latter is more commonly used in professional and scientific contexts.

Healthspan extension becomes increasingly critical as nations evolve into “ageing”, “aged”, and “super-aged” societies, where older populations represent over 7%, 14%, and 20% of the total population, respectively [6]. Despite being a relatively young nation, Singapore rapidly transitioned to an “ageing” society within four decades of independence, achieving “aged” status by 2021 [7]. Projections indicate that by 2030, over a quarter of Singapore’s population will be 65 years or older, placing it among “super-aged” nations, a demographic trend observed in countries such as Japan, Italy, Finland, and Korea. This demographic shift in Singapore is largely driven by a declining total fertility rate (TFR), which fell to 1·04 births per woman in 2022, far below the replacement rate of 2.1 [8]. Concurrently, the old-age dependency ratio—representing the number of dependents per 100 working-age individuals—has risen from 8.5 to 20.7 over the past two decades [7].

The shift in demographic age distributions has coincided with a high prevalence and projected rise in ARDs which were linked to over 80% of Singapore’s disability-adjusted life years (DALYs) [9] and over 60% of global mortality in 2019 [10]. In the same year, global life expectancy was recorded at 73.0 years [7], while health-adjusted life expectancy (HALE), or healthspan (the period of life spent in good health), was 63.7 years [10]. Singapore ranked second globally for life expectancy at birth, at 84.9 years (just behind Hong Kong at 85.2 years), and led the world in HALE with 74.5 years in 2019. Over the past two decades, a widening gap between lifespan and healthspan has become evident in many ageing nations [10].

With a shrinking younger population and an expanding older demographic, healthcare expenditures are projected to triple between 2010 and 2030 [11], raising concerns about long-term sustainability. For context, the USA allocated 18.8% of its gross domestic product (GDP) to healthcare in 2019—the highest globally at the time—yet faced persistently high mortality due to non-communicable diseases, at 88% over the previous two decades, while improving its health-adjusted life expectancy (HALE) by just over 1 year [7]. In contrast, Singapore spends approximately 4% of its GDP on healthcare while maintaining comparable health outcomes with other developed ageing nations [12]. Direct investments aimed at slowing ageing or extending life expectancy by a single year have demonstrated superior economic outcomes, with annual benefits equivalent to 4–5% of GDP, amounting to approximately US$38 trillion [13, 14]. Although transitioning to a new model of care may incur higher initial costs, it has the potential to generate significant long-term financial savings when scaled. This is exemplified by the “Osana experiment” in Australia, where a focus on proactive care, patient engagement, and data-driven approaches in primary care has resulted in improved health outcomes [15].

## Optimising health-adjusted life expectancy

In response to the growing challenges of an ageing population, Singapore has been actively reshaping its public healthcare system to prioritise healthy ageing, aiming to manage healthcare costs, retirement income, long-term care, and the adoption of new medical technologies [16]. By delaying the adverse effects of ageing, extending healthspan, and shifting towards preventive health interventions, Singapore seeks to harness the benefits of an ageing population to drive economic growth, potentially achieving a second demographic or longevity dividend [17]. Singapore envisions itself as a “Blue Zone 3.0,” where preventive health interventions extend the population’s healthspan, building on its status as an engineered “Blue Zone 2.0” [18]. This distinction reflects the success of policies that integrate health considerations into urban planning, making Singapore one of the most liveable cities in the world [19].

In 2018, Singapore reorganised its public healthcare system into three integrated clusters, designed to enhance the delivery of person-centred care across primary, secondary, and tertiary care levels, as well as intermediate and long-term care, while emphasising prevention and health promotion. The three healthcare clusters include—the National Healthcare Group (NHG), Singapore Health Services (SingHealth), and the National University Health System (NUHS). These clusters helped prepare for Singapore’s major preventive care strategy, *Healthier SG*, which was launched by the Ministry of Health (MOH) in July 2023 [20, 21]. The strategy aims to empower Singaporeans to take proactive control of their health by leveraging a robust network of over 1800 private general practices that manage 80% of Singapore’s primary healthcare, of which 712 GP clinics were enrolled as of November 2024 [9, 22, 23]. *Healthier SG* offers free screenings, vaccinations, and access to health programs, along with subsidised assessments and medications aimed at preventing ARDs. The program strengthens care coordination by connecting patients with community partners for health and social support while enabling health tracking through mobile applications [20]. Community integration is central to the program, with healthcare clusters coordinating regional services, community partners offering health-promoting activities, and active ageing centres delivering programs recommended by general practitioners (GPs) and regional health clusters [24].

In the eastern region, SingHealth has set up teams of community nurses, well-being coordinators, primary care providers, and community partners across more than 30 locations [25]. The SingHealth Community Hospitals Office of Learning (SCHOOL) has trained over 1000 professionals in social prescribing practices and was designated as the World Health Organization’s first Collaborating Centre for Social Prescribing [24].

In the central-north region, NHG focused on building robust community health teams that include nurses, allied health professionals, and health coaches. These teams work alongside GPs and community partners to deliver comprehensive care [25]. To prepare GPs for this shift, NHG’s Primary Care Academy (NHGP) developed a training program featuring workshops and online modules on preventive care, behavioural counselling, and team-based care approaches. Clinic assistants are also included in the training to ensure cohesive support within the primary care networks (PCNs) [25].

In the western region, NUHS has been actively engaging with GPs, building a network of more than 130 practitioners, and increasing the number of community health posts, which currently exceed 30, serving as local hubs where residents can access information about health services available near their homes [25].

**Fig. 1:**
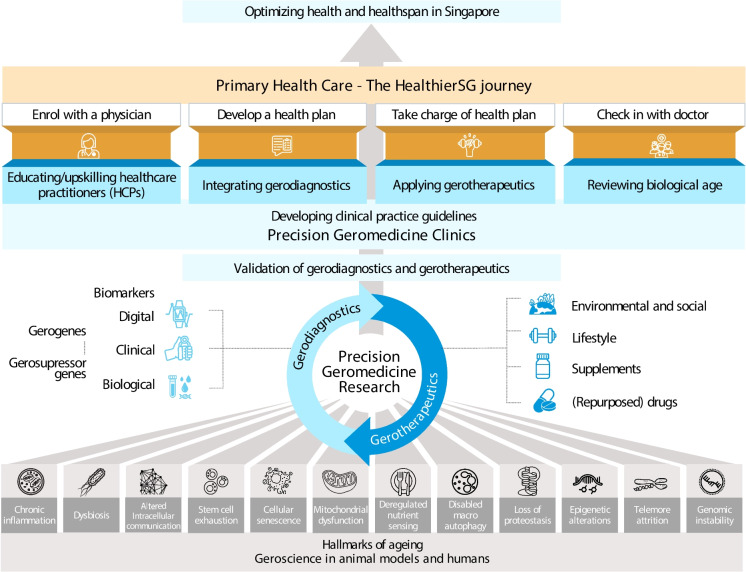
Augmenting public health initiatives for healthy ageing with Precision Geromedicine

Along with *Healthier SG*, Singapore is advancing research in geroscience, a field that connects the biology of ageing with the biology of disease [26]. This research seeks to explore how emerging clinical innovations can augment healthy ageing initiatives, further strengthening Singapore’s commitment to optimising healthspan and preventing the onset of ARDs by developing Precision Geromedicine (Fig. 1).

## Towards Precision Geromedicine

Geroscience is an emerging field focused on understanding the biological mechanisms of ageing, conceptualised as hallmarks of ageing [3], and their contribution to ARDs, such as diabetes, cardiovascular disease, neurodegenerative disorders, and cancer. It posits that ageing is the primary risk factor for many ARDs and aims to develop gerotherapeutic interventions for the prevention and treatment of ARDs to extend both lifespan and healthspan [4]. This gives rise to the emergence of geromedicine, a new proposed evidence-based medical speciality focused on optimising health and preventing ARDs by targeting the ageing process itself in ageing adults. While ageing is not officially classified as a disease and cannot be treated as such in clinical trials approved by regulatory bodies like the U.S. Food and Drug Administration (FDA) or European Medicines Agency (EMA), research predominantly targets the treatment or, less commonly, the prevention of multimorbidity and specific ARDs [27]. However, the World Health Organization (WHO), in its International Classification of Diseases 11 th Revision (ICD-11), defines “ageing-associated decline in intrinsic capacity” as a disease category. Intrinsic capacity refers to the total physical and mental capacities an individual can draw upon, reflecting vitality across multiple physiological systems, such as energy metabolism and immune function [28]. Functional ability often declines with age, and ageing-related conditions—such as diabetes, hypertension, and hypercholesterolemia—frequently precede this decline. Early mitigation of these risk factors is crucial to preserving organ function and overall functional capacity, which is what geromedicine aims to achieve. This framework of intrinsic capacity can be integrated into Singapore’s healthy ageing initiatives.

### Gerodiagnostics

Biological ageing is a process influenced by environmental factors and accelerated by the activation of gerogenes and the inactivation of gerosuppressive genes [4]. In contrast to the hallmarks of ageing concept, which encounters methodological, quantitative, and validation challenges, a genetics-based perspective on ageing benefits from readily accessible methodologies, such as genomic, epigenomic, and postgenomic techniques. It allows for the establishment of objective associations between genotype, expression of gerogenes and gerosuppressor genes, and the phenotype.

Changes in the expression of gerogenes and gerosuppressor genes can be assessed indirectly through the epigenome, typically measured by DNA methylation patterns. Analyses of these data have led to “epigenetic clocks”, which estimated biological age calibrated based on chronological age (first-generation clocks), mortality risk (second-generation clocks), or ARDs (third-generation clocks) [2]. These clocks can reveal deviations in an individual’s ageing trajectory relative to a reference population and are being validated in observational and interventional studies as predictors of adverse health outcomes [29, 30].

Gene expression can also be assessed at the mRNA or more often used, protein level. Plasma proteomics, which reflects proteins from all tissues, allows for the examination of organ-specific ageing processes [31]. This approach has highlighted variability in the pace of ageing both between individuals and within different organ systems [31]. A cross-validated plasma proteomic ageing clock has been shown to predict ageing-related functional status, multimorbidity, and mortality risk in diverse populations [32].

These omics-based approaches are generating a growing number of biological biomarkers of ageing that have great clinical potential once validated. Alongside biological markers, clinical and digital biomarkers are already being used in clinical practice to optimise health. Organ-specific clinical clocks, based on parameters such as lung function, cardiac health, and musculoskeletal measures, predict mortality and the onset of ARDs in healthy middle-aged individuals and reveal patterns of multi-organ ageing [33]. Digital health technologies such as remote monitoring [34] have the potential to transform healthcare by providing insights into physiological, behavioural, and environmental factors, which can be used as predictors or support real-time behavioural interventions [35].

Although the ideal situation for clinical practice would involve assessing ageing with a single biomarker of ageing, the variability in ageing rates among individuals complicates this approach. Thus, there is growing support for the development of composite biomarkers for gerodiagnostics, as recommended by the Biomarkers of Ageing Consortium [36].

### Gerotherapeutics

Recent advancements have facilitated the translation of gerotherapeutic strategies from animal models to human applications. The Healthy Longevity Medicine Society [37] identified four key categories of interventions: lifestyle, social/environmental, supplements, and drug repurposing [38].

Lifestyle interventions, including diet, exercise, stress management, sleep, social connections, and avoiding harmful substances, form the foundation of health optimization. Given the robust evidence supporting lifestyle modifications, personalised approaches based on biomarkers of ageing are essential for maximising their efficacy. Social and environmental interventions, such as community engagement, social support networks, and cognitive and physical training programs, are vital for improving physical function, cognitive performance, and overall quality of life. These strategies help foster an environment conducive to healthy ageing.

Supplement-based interventions are gaining attention due to their potential to improve lifespan and healthspan. Compounds like nicotinamide mononucleotide (NMN) [39], alpha-ketoglutarate (AKG) [40], glycine [41], senolytics (e.g., fisetin and the dasatinib-quercetin combination) [42], urolithin A [43], and spermidine [27], have shown promise in preclinical studies and early human trials. Drug repurposing is another key strategy in geromedicine, focusing on FDA-approved medications with potential geroprotective effects. Notable examples include diabetes drugs [44] such as sodium-glucose cotransporter-2 (SGLT2) inhibitors, metformin, and acarbose, as well as immunosuppressants such as rapamycin and its analogues [45]. Potentially emerging treatments, including autologous platelet-rich plasma, exosome, or stem cell injections, have not yet undergone rigorous human clinical trials to confirm their effects on ageing and longevity in humans.

Overall, although the above gerotherapeutic interventions exhibit promising potential, further research is necessary to evaluate their effectiveness in healthy individuals, a priority for ongoing studies at the Yong Loo Lin School of Medicine.

### Towards clinical implementation of geromedicine

The field’s growth has been driven by increasing societal interest, expanded funding, and the rise of biotech companies exploring solutions to optimise health, with the potential to transform how ARDs are being addressed by targeting ageing itself. Recognising its growing older population, Singapore has emerged as an early adopter, progressively developing its healthy longevity ecosystem through various longevity R&D centres, non-governmental organisations, companies, and investments in translational geroscience research [46]. These investments have expanded healthy ageing research in Singapore over the past two decades [47]. National programs, such as the Healthy Longevity Global Grand Challenge by the National Medical Research Council have helped build a strong research capacity for its growth, and more clinical trials are underway to translate geroscience into human research and eventually into clinical practice.

The establishment of “longevity clinics” adhering strictly to evidence-based medicine is crucial. Ideally, these clinics should be integrated within publicly funded hospitals, embedding this new speciality into existing healthcare and educational services to make longevity-focused care more accessible to the public [38]. In 2023, a healthy longevity clinic was launched within the publicly funded National University Health System (NUHS), Alexandra Hospital in conjunction with Sheba Medical Centre in Israel, and the Mayo Clinic in the USA [38]. However, while these clinics represent an innovative clinical model, the evidence regarding their feasibility and effectiveness in public healthcare settings is still emerging. Alexandra Hospital’s Well programme is implementing a new care model aimed at evaluating participants’ acceptability of longevity services, recruitment, retention, and adherence. This comprehensive evaluation will also assess the cost-effectiveness of provided gerodiagnostics and gerotherapeutics. Ultimately, this process will contribute to building the evidence base for developing clinical practice guidelines, a task led by the Healthy Longevity Medicine Society [5, 37], which promotes interdisciplinary collaboration and high standards of clinical care.

To ensure that research findings are ethically consolidated and widely disseminated, the NUS Academy for Healthy Longevity was founded to create standardised training and educational programs for all stakeholders [48]. The NUS Academy for Healthy Longevity aims to bridge the skill gap in integrating Precision Geromedicine into healthcare. It focuses on evidence-based, standardised training programs to promote ethical practices and share research findings. The Academy aims to enhance the skills of diverse stakeholders, including the public, healthcare professionals, related industries, and policymakers, through evidence-based education. It offers public seminars to raise awareness and targeted courses to advance professionals’ expertise. Furthermore, an accredited master’s program in Precision Geromedicine is being developed to provide a rigorous curriculum and prepare future leaders in the field.

Furthermore, the international Healthy Longevity (HELO) consortium [49], which includes eight countries, is actively exploring the public attitudes towards lifespan, healthspan, and geromedicine. The framework includes components assessing awareness and knowledge; the former measures subjective importance attributed to geromedicine-related concepts, while the latter evaluates the public’s understanding of these concepts. The motivation component examines factors influencing one’s decision to pursue healthy longevity, which are further categorised into five domains: personality, current behaviours, personal values, views and beliefs, and social norms. This initiative together with the McKinsey Health Institute’s global survey [50] and the Hevolution’s global healthspan report will inform researchers and policymakers about the needs and perceptions of the general population. The McKinsey Health Report underscores the benefits of older adult participation in society, finding that those engaged in work, volunteering, or lifelong learning report a 4–8% better health status [50]. Additionally, the Hevolution Global Healthspan Report highlights discussions on ageing research, healthspan, and innovative healthcare strategies, focusing on geroscience, biomarkers, and interventions. It suggests the need for better gerodiagnostics, the importance of personalised gerotherapeutics, and the economic and ethical implications of healthspan [51].

## Leveraging public health initiatives in Singapore

As evidence continues to accumulate, validated tools will increasingly enable clinicians to address the underlying causes of ARDs by understanding a patient’s ageing processes through gerodiagnostics. These tools will allow healthcare providers to develop comprehensive, personalised management plans using gerotherapeutics. Preventive programs that have already been implemented, such as those introduced through *Healthier SG*, which reduce out-of-pocket costs, can further support the integration of gerodiagnostics into routine care. Additionally, current digital health platforms such as *HealthHub* and *Healthy365* can be leveraged to boost enrolment and engagement, reinforcing positive health behaviours [52]. For instance, the *National Step Challenge*, the world’s first population-level fitness tracker initiative, led to a 9% increase in incidental physical activity and a 5% rise in moderate-intensity activity among participants from years 2010 to 2017 [52]. The program’s success was driven by the *Easy, Attractive, Social, and Timely (EAST)* framework, which promotes physical activity through nudging and gamification [52]. Given the widespread use of these platforms, clinicians in Precision Geromedicine can also utilise patients’ digital biomarkers of ageing to tailor personalised gerotherapeutic interventions [2].

Gerotherapeutics can further capitalise on Singapore’s well-designed urban environment, fostering social prescribing. Since 1967, green spaces have proliferated under initiatives such as the *Garden City* program, which aims to achieve a park provision ratio of 0.8 ha per 1000 people by 2030, ensuring that at least 80% of households have access to green spaces. In 2019, over 80% of Singaporeans resided in public housing flats developed by the Housing & Development Board (HDB) across various districts [19]. These districts are designed to provide walkable access to essential amenities such as transportation, food, and healthcare while promoting family-centred living—a design that surpasses other age-friendly cities, where diverse housing policies have benefited fewer than 0.3% of older residents [53]. Design initiatives such as these are in line with the WHO age-friendly cities framework together with The United Nations Decade for Healthy Ageing, which serve to identify and address environmental barriers for older people. Building on this, the Urban Development Authority in Singapore is envisioning a long-term plan through the Draft Master Plan 2025 to translate initiatives into detailed land use and development for the next 10 to 15 years [54].

These commitments to successful ageing through built environment are further outlined in the 2023 *Action Plan for Successful Ageing*, which includes other enablers such as provisions for wealth accumulation, training and education opportunities, and raising the retirement age [16]. Singapore is uniquely positioned to implement and evaluate these enablers at a national level, making it a leader in the global push for healthy ageing.

## Governance and AgeTech in Singapore

In 2021, Singapore ranked in the 100 th percentile for government effectiveness, outperforming other ageing nations such as Switzerland (99), Denmark (98.6), Finland (98.1), Hong Kong (93.3), and Japan (89) [7]. Multisectoral collaboration among various government bodies—including the Ministry of Health (MOH), which oversees the Health Promotion Board and Health Sciences Authority, alongside the Ministries of Manpower, Finance, and Technology—is essential for fostering the socio-political environment necessary to implement Singapore’s ambitious ageing action plan. For example, the Ministry of Health Office for Healthcare Transformation (MOHT) has been reshaping Singapore’s healthcare system through five key programs [55]. Future primary care (FPC) empowers patients to manage chronic diseases via telehealth and home technologies; Integrated general hospital (IGH) improves coordination between hospitals and community settings; Integrated health promotion (InHealth) focuses on disease prevention; Data, science and technology (DST) utilises innovative technologies and data to enhance healthcare outcomes; and communications and engagement (C&E) strengthens community outreach. MOHT is collaborating with partners to design initiatives and develop enablers such as the Healthier SG Implementation Office (HSI), which works with various stakeholders to develop care protocols and drive the Healthier SG objectives. Additionally, the National Improvement Unit (NIU) is leading quality improvement initiatives such as the National Diabetes Collaborative and supports Primary Care Networks (PCNs) with quality improvement projects aligned with Healthier SG goals.

Building on this strong governance infrastructure, Singapore’s MedTech sector has seen significant growth in research and development since the 1990 s. The Economic Development Board (EDB), A*STAR, and various ministries, through the National Science and Technology Board, have worked together to establish Singapore as a major biomedical sciences hub. This has led to the development of key facilities such as Tuas Biomedical Park, Biopolis, and MedTech Hub, contributing to an increase in manufacturing output from S$3 billion to S$18·7 billion by 2022[56]. The number of biomedical startups also surged from 59 in 2010 to over 300 by 2020. Singapore’s global reputation as a top destination for business—ranked second in the world for ease of doing business, first in credit rating, and third in e-penetration [7, 57]—has helped foster an inclusive and well-regulated digital environment conducive to cross-sector collaborations. One notable success in this space is Illumina, a precision health company that supplies over 90% of its global microarray demand and produces more than 60% of its DNA sequencing consumables, including reagents and kits, at its Singapore facility[56]. Additionally, the PRECISE-SG100 K research program is set up to determine behavioural, environmental, genetic, and biological factors underlying diseases prevalent in the multi-ethnic Asian population of Singapore. This can help clinicians in Precision Geromedicine to provide personalised health plans through the Healthier SG initiative, leveraging health-tracking apps to enhance precision public health [58]. With this strong foundation, Singapore is well-positioned to lead advancements in gerodiagnostics and gerotherapeutics within the Precision Geromedicine field [2].

This growth, however, will depend on effective data sharing between public and private healthcare settings. Ranked third globally for digital competitiveness, Singapore’s strong digital readiness, connectivity, and data interoperability are further bolstered by its centralised National Electronic Health Record (EHR) system [57] and the Next Generation Electronic Medical Record (NGEMR) [59]. The NGEMR is a multi-year initiative designed to integrate care delivery and patient management across the various health clusters. This modern, centralized EMR system is replacing nearly 90 legacy systems at NHG and NUHS, streamlining workflows, data, and reporting [59].

This robust infrastructure enables innovations such as large-scale decentralised observational and interventional research, and the creation of comprehensive datasets accessible to multidisciplinary healthcare teams, paving the way for further advancements in AgeTech and multidisciplinary healthcare delivery.

## Challenges and future of Precision Geromedicine in Singapore

Transitioning healthcare from a reactive, treatment-focused approach to one that prioritises upskilling and education to optimise healthspan offers, a more sustainable solution for addressing the challenges of an ageing population. However, *Precision Geromedicine* is still in its nascent stages and has not yet been integrated into healthcare systems [38]. The field is currently working toward the development of the scope of practice and clinical practice guidelines to help multidisciplinary clinicians deliver evidence-based care while adhering to ethical and medico-legal standards.

A critical challenge lies in the need to establish standardised datasets for applied gerodiagnostics and gerotherapeutics. The assessment of ageing biomarkers requires the development and validation of novel diagnostic tools that are not yet available in routine clinical practice [60].

Importantly, the deployment of precision gerotherapeutics needs to be calibrated to established ethical and legal requirements around informed consent, shared decision-making, and equity of access. Given that age carries with it much personal investment and is socially sensitive, the ethically appropriate disclosure and management of a patient’s biological age can pose difficulties. Such challenges highlight the need for implementation science and interdisciplinary collaborations with academic and research institutions. In addition, public awareness and engagement are vital for the successful adoption of Precision Geromedicine. Education campaigns and professional training for healthcare providers will be essential to foster acceptance and understanding of these new approaches, both among the public and within the healthcare sector. Addressing concerns about data privacy, informed consent, and the potential risks of personalised gerotherapeutics will require ongoing dialogue and the development of clear, transparent policies within different healthcare settings. Nevertheless, in Singapore, health information is regulated by a single, well-established data protection statute alongside common law duties of confidentiality. This requires health data, such as that collected in HLM clinics, to follow standard procedures for protecting sensitive data and maintaining medical confidentiality. The forthcoming Health Information Bill [61], due to be enacted in 2025, will further strengthen the protection of health data. This new law places a positive legal obligation on health providers to record health information on the National Electronic Health Record and to share that information between themselves for a limited number of purposes that advance patient care, as well as mandating a strengthening of cyber security and the governance of data protection systems. For research into HLM, the Human Biomedical Research Act strictly limits the use of data collected for research purposes, found in informed consent requirements and overseen by institutional review boards. In the face of ongoing complexity, this robust legal framework is also strengthened by well-established ethics and law education programs in health care training as well as new mandatory continuing professional development courses in ethics and law for medical doctors that are designed to cascade good practice around confidentiality and data protection throughout the health system.

Singapore, with its well-structured healthcare financing and supportive socio-political environment, is well-positioned to facilitate the integration of Precision Geromedicine into its public healthcare system. By gradually building capacity and leveraging its strengths in biomedical research and innovation, Singapore can transform healthcare delivery and emerge as a global leader in optimising both the health and healthspan of its population. This strategic positioning not only enhances the nation’s healthcare infrastructure but also solidifies its role as a hub for cutting-edge Precision Geromedicine research and application.

## Data Availability

Not applicable.
